# Surgical treatment of recurrent intussusception induced by intestinal lymphoid hyperplasia in a child: is bowel resection always necessary? A case report

**DOI:** 10.1186/s12893-022-01608-w

**Published:** 2022-05-10

**Authors:** Hui Wang, Hongyan Li, Wang Xin, Liandi Xu, Guoqing Zhang, Qingtao Yan

**Affiliations:** 1 Department of Dermatology, Weifang People’s Hospital, Weifang, 261041 China; 2 Department of Endocrinology, Weifang People’s Hospital, Weifang, 261041 China; 3 Department of Pediatric Surgery, Weifang People’s Hospital, 151 Guangwen St, Kuiwen District, Weifang, 261041 China; 4 Department of Ultrasound, Weifang People’s Hospital, Weifang, 261041 China

**Keywords:** Case report, Intussusception recurrence, Intestinal lymphoid hyperplasia, Enema reduction, Bowel resection

## Abstract

**Background:**

Intussusception recurrence (IR) induced by intestinal lymphoid hyperplasia (ILH) in children is rare, and surgical treatment is the final resort if IR is refractory to medications and non-surgical interventions. To date, only a few case reports have described surgical management of ILH-induced IR in children, all involving bowel resection regardless of whether there are bowel necrosis and perforation.

**Case presentation:**

A 2-year-old boy was transferred to our department due to IR. His main complaint was abdominal pain. Color Doppler ultrasound confirmed ileocecal intussusception while no other abnormalities were found. A final diagnosis of IR with unknown causes was made. Repeated saline enema reductions and dexamethasone failed to cure the IR. Laparotomy was eventually performed after almost 10 episodes of IR. Intraoperatively, distal ileum thickening with palpable masses without bowel necrosis and perforation was noted. ILH was suspected and a biopsy of the affected intestine was performed. Histopathological analysis confirmed ILH. The intussusception was manually reduced, the terminal ileum and the ileocecal junction were fixed to the paralleled ascending colon and the posterior peritoneum respectively, and no bowel resection was performed. The postoperative recovery was uneventful and no IR was observed during over 5 years of follow-up.

**Conclusions:**

As far as we are aware, this is the first report of successful surgical treatment of ILH-induced pediatric IR without bowel resection in a child. Our experience suggests bowel resection may be unnecessary if bowel necrosis and perforation are absent.

## Background

Intussusception occurs when a portion of intestine folds into the lumen of an adjacent bowel segment, which mostly affects children under the age of 3 years [1]. Both non-operative and operative interventions are effective in the management of intussusception [1, 2]. Nevertheless, intussusception recurrence (IR) in children is not uncommon [3–5]. A recent meta-analysis has shown that presence of fever and a pathological lead point are associated with IR in children [3]. Intestinal lymphoid hyperplasia (ILH) is an uncommon cause for IR in children [6]. Currently, only a few case reports have described surgical management of ILH-induced IR in children, all involving bowel resection regardless of whether there are peritonitis and bowel perforation [7–10]. We describe here for the first time, to our knowledge, the successful surgical treatment of ILH-induced IR without bowel resection.

## Case presentation

A 2-year-old boy presenting with paroxysmal abdominal pain for 1 day was diagnosed with ileocecal intussusception and treated with saline enema in a local hospital in December, 2015. He was transferred to our department due to IR the following day. At admission, the patient’s main complaint was abdominal pain without vomiting, hematochezia or melena. History of diarrhea, food allergy, genetic disease and chronic disease was denied. Physical examination revealed normal vital signs. Abdominal tenderness without palpable masses was noted. Routine blood testing and rotavirus antibody measurement were negative. Abdominal CT scan revealed no abnormalities. Color Doppler ultrasound confirmed ileocecal intussusception. Final diagnosis of IR with unknown causes was made.

**Fig. 1:**
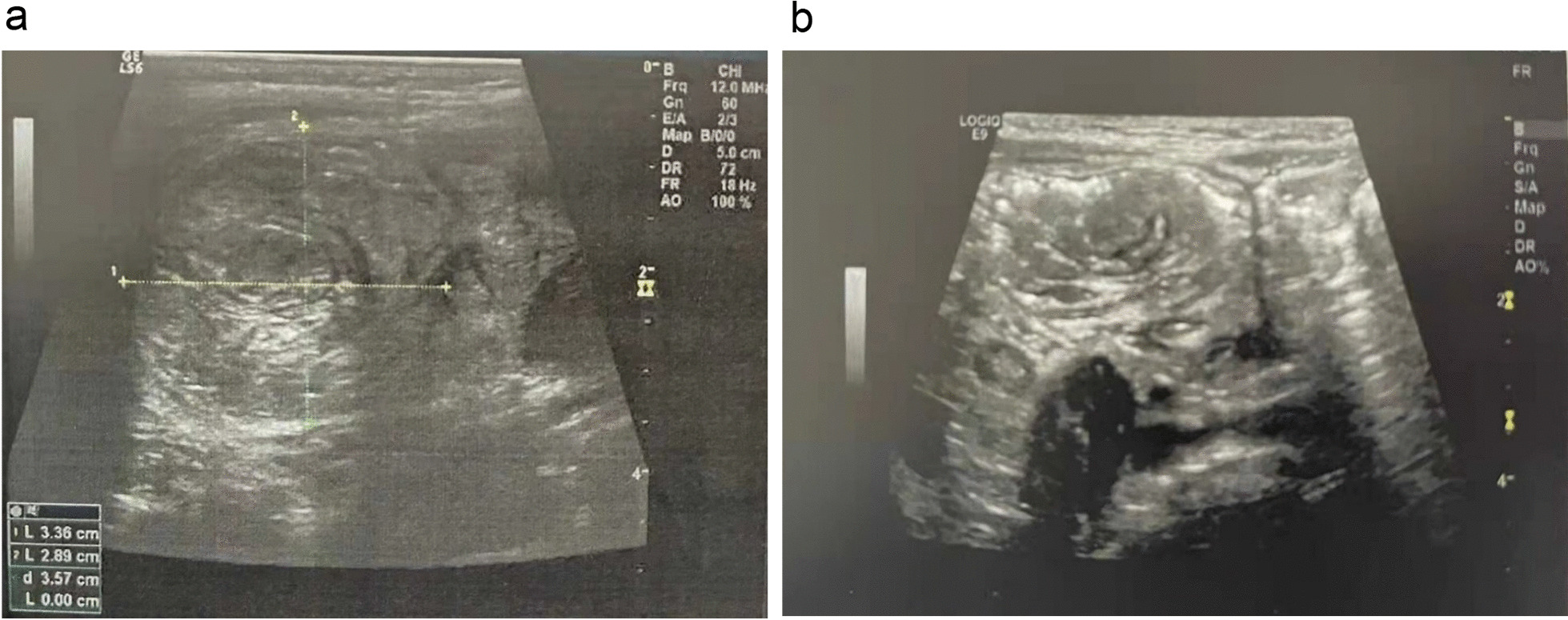
Ultrasound examination before and after enema reduction. A hypoechoic ring was seen (panel **a**) by ultrasound, a feature of intussusception, before enema reduction, which disappeared after reduction (panel **b**)

**Fig. 2:**
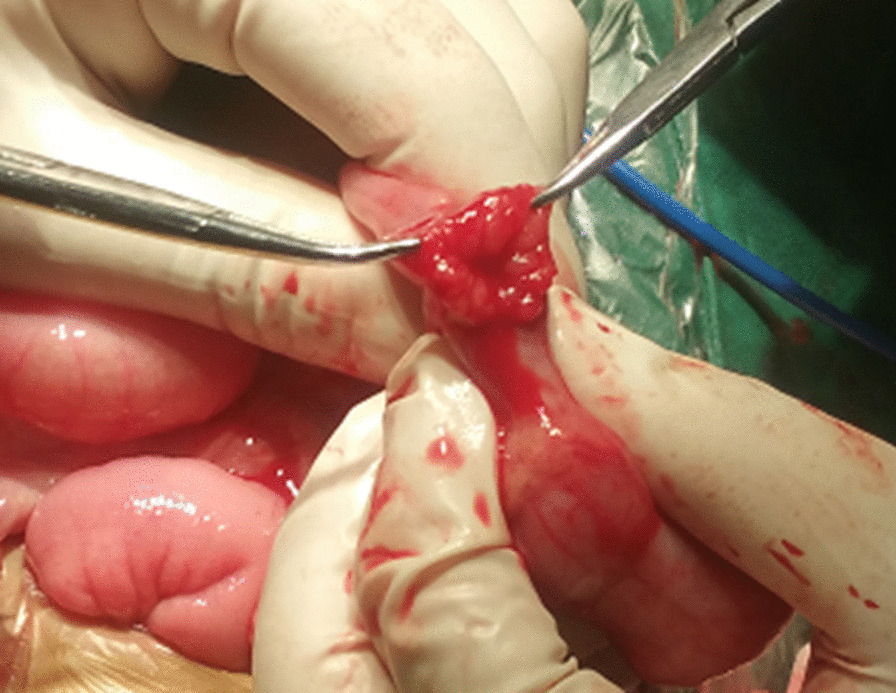
Intraoperative exploration of the distal ileum. The distal ileum was opened and scores of nodular mucosal protrusions were seen

**Fig. 3:**
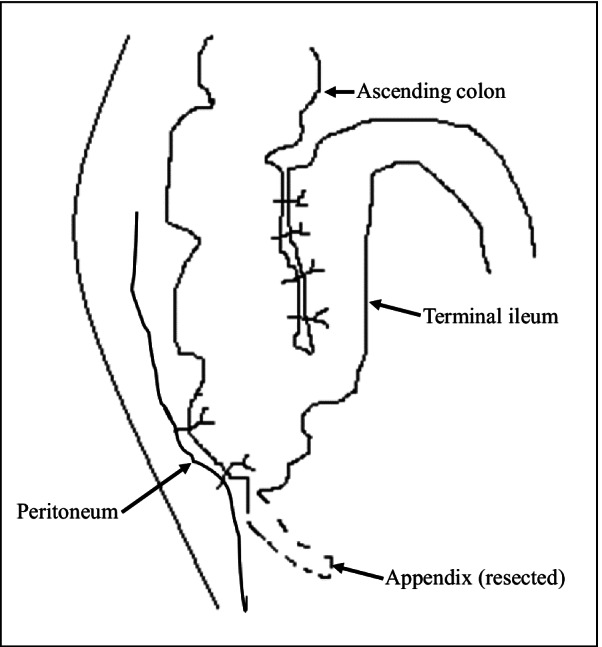
Surgical techniques. A schematic diagram shows the fixation of the terminal ileum to the paralleled ascending colon, and the ileocecal junction to the peritoneum. The appendix was resected

**Fig. 4:**
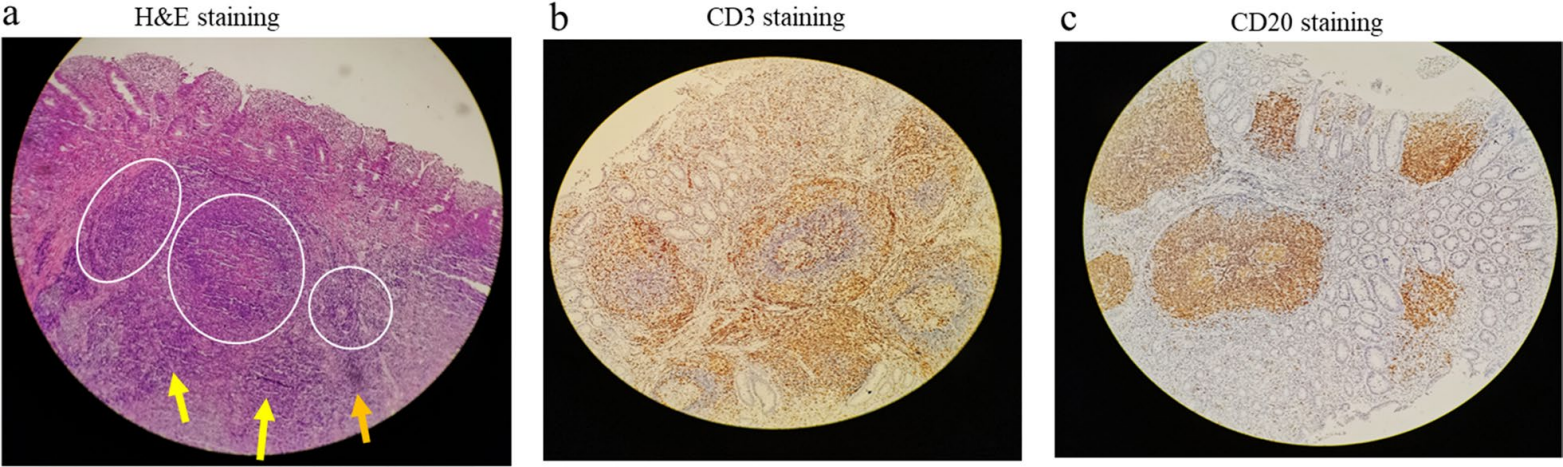
Histopathological results. H&E staining showed hyperplastic diffuse lymphoid tissue (yellow arrows) and lymphoid follicles (circled) in the biopsy sample (panel **a**). CD3 (+) T lymphocytes (stained brown, panel **b**) were seen in both the diffuse lymphoid tissue and lymphoid follicles, while CD20 (+) B lymphocytes (stained brown, panel **c**) were distributed predominantly in lymphoid follicles

Ultrasound-guided saline enema was performed and intussusception was successfully reduced. The patient was administered with dexamethasone (3 mg) intravenously. The following day the child encountered another episode of intussusception, and saline enema was repeated, resulting in the reduction of the intussusception. Intravenous dexamethasone was continued. During the period of day 3 to day 8, intussusception recurred almost every day with no signs of peritonitis and perforation, or ultrasound findings of intestinal ischemia, trapped fluid and bowel obstruction. The parents of the patient were informed of different treatment alternatives including surgery, but all episodes of intussusception were treated with saline enema while intravenous dexamethasone was discontinued on day 5. Complete reduction of the intussusception was confirmed by Ultrasound after each saline enema (Fig. 1). When intussusception recurred on day 9, in view of that multiple saline enema reductions and dexamethasone failed to cure the IR, laparotomy was eventually performed upon the consent of the parents, which revealed ileocecal intussusception without bowel necrosis and perforation. The intussusception was manually reduced, and distal ileum thickening with palpable masses were noted. The distal ileum was opened for exploration with a 1 cm longitudinal incision, and scores of nodular mucosal protrusions were seen (Fig. 2). ILH was suspected and a biopsy of the affected intestine was performed. Afterwards, the incision was closed by interrupted inverted transmural plus interrupted seromuscular stitches using a 5-0 absorbable suture. The terminal ileum (~ 6 cm in length), and the ileocecal junction were fixed to the paralleled ascending colon and the peritoneum by interrupted seromuscular stitches using a 4-0 silk suture, respectively (Fig. 3). The appendix was removed. Histopathological analysis of the surgical biopsy revealed hyperplastic diffuse lymphoid tissue and lymphoid follicles (H&E staining) (Fig. 4a) with abundant CD3 (+) T lymphocytes (Fig. 4b) and CD20 (+) B lymphocytes (Fig. 4c). It appeared that B lymphocytes were distributed only in lymphoid follicles while T lymphocytes were seen in both the diffuse lymphoid tissue and lymphoid follicles. The postoperative recovery was uneventful and no IR was observed during over 5 years of follow-up.

## Discussion and conclusions

ILH, a result of enhanced immune response to foreign antigens such as virus, bacteria and food components in young children [11], is a rare cause for IR [6]. A few case reports have shown that corticosteroids are effective for the treatment of ILH-induced IR in children [12–14]. In contrast, we observed that IR was not responsive to dexamethasone. The reason for this failure remains unknown.

Adenovirus and rotavirus have long been recognized as potential infectious agents for intussusception in children [15]. In the present case, rotavirus antibody was negative, however, adenovirus as the cause of IR could not be excluded as adenovirus testing was not performed. Food allergy induced-ILH as the cause of IR has been described [8], but family history of food allergy was denied in the present case.

Currently, only a few case reports have described surgical intervention of ILH-induced IR in children, all involving bowel resection [7–10]. Hasegawa et al. successfully treated a 6-year-old boy with a surgical procedure involving ileocecal resection and fixation of the ileum to the retroperitoneum [7]. In another case study, a female infant who experienced 7 episodes of intussusception associated with ILH eventually underwent ileocecal resection and right hemicolectomy [8]. Ileocecal resection and right hemicolectomy were also performed by Jona et al. in two children and by Cornes and Dawson in a male infant for the treatment of ILH-induced IR [9, 10]. Bowel resection was performed in all of these cases, despite that some cases were not complicated by bowel necrosis and perforation [7, 8]. In contrast, we did not perform bowel resection in view of the absence of bowel necrosis and perforation, and post-operative IR was not observed in our patient during over 5 years of follow-up. Of note, the follow-up time was not clearly indicated in previous case reports. [7–10].

An advantage of small intestine fixation is that it avoids anastomotic leakage, a severe complication after bowel resection. However, it has been shown that bowel resection results in a lower rate of recurrence [16]. Koh et al. reported that none of 29 children undergoing bowel resection had recurrence. In contrast, those with ileopexy had a higher recurrence rate (4%, 8 out of 186), although the recurrence rates in the two groups were not significantly different [16]. It is speculated that bowel resection removes the segment responsible for intussusception, thereby preventing recurrence [16]. Although at a very low rate, intestine adhesion and obstruction can occur after either laparoscopic or open surgical reduction of intussusception [17–19]. However, direct comparison of this complication in children with simple surgical induction and those with surgical induction plus ileopexy has not been described. A recent study revealed a case of small bowel obstruction in a total of 100 children who had laparotomy without ileopexy [18]. In contrast, in another recently published study, it was shown that no small intestine adhesion or obstruction occurred in a total of 61 children who had ileopexy [19].

Although a classic “rule of threes” recommends the number of enema reduction attempt be capped at three [15], many clinicians have discarded this rule and used nearly unlimited number of attempts as reviewed by del-Pozo et al. [20] In our practice, if consent to a surgical protocol is not obtained and the contraindications are absent, edema induction will be repeated for IR. For the present case, enema reduction along with steroid medication failed to cure the IR, and surgery became the last resort.

In conclusion, this case report provides two important tips regarding the diagnosis and treatment of ILH-induced IR in children. First, if imaging and other exams are unable to determine the cause of IR in children, ILH should be highly suspected, and biopsy pathology is the key for the identification of ILH. Secondly, when medications and non-surgical interventions fail to cure IR, bowel resection may be avoided during surgical procedures if bowel necrosis and perforation are absent. We hope this case report will spur more clinicians to share their experiences in surgical treatment of ILH-induced IR without bowel resection in cases similar to ours.

## Data Availability

All data and materials are included in the article.

## Abbreviations

IR: Intussusception recurrence
ILH: Intestinal lymphoid hyperplasia
CT: Computerized tomography

## References

[1] Savoie KB, Thomas F, Nouer SS, Langham MR, Huang EY (2017). Age at presentation and management of pediatric intussusception: a Pediatric Health Information System database study. Surgery.

[2] Gluckman S, Karpelowsky J, Webster AC, McGee RG. Management for intussusception in children. Cochrane Database Syst Rev. 2017;6(6):CD006476. 10.1002/14651858.CD006476.pub3 PMC6481850 28567798

[3] Ye X, Tang R, Chen S, Lin Z, Zhu J (2019). Risk factors for recurrent intussusception in children: a systematic review and meta-analysis. Front Pediatr.

[4] Hsu WL, Lee HC, Yeung CY, Chan WT, Jiang CB, Sheu JC (2012). Recurrent intussusception: when should surgical intervention be performed?. Pediatr Neonatol.

[5] Niramis R, Watanatittan S, Kruatrachue A, Anuntkosol M, Buranakitjaroen V, Rattanasuwan T (2010). Management of recurrent intussusception: nonoperative or operative reduction?. J Pediatr Surg.

[6] Navarro O, Daneman A (2004). Intussusception: part 3: diagnosis and management of those with an identifiable or predisposing cause and those that reduce spontaneously. Pediatr Radiol.

[7] Hasegawa T, Ueda S, Tazuke Y, Monta O, Sakurai T, Takahara N (1998). Colonoscopic diagnosis of lymphoid hyperplasia causing recurrent intussusception: report of a case. Surg Today.

[8] Masilamani K, Jolles S, Huddart S, Tuthill DP (2009). Successful dietary treatment of recurrent intussusception. Arch Dis Child.

[9] Jona JZ, Belin RP, Burke JA (1976). Lymphoid hyperplasia of the bowel and its surgical significance in children. J Pediatr Surg.

[10] Cornes JS, Dawson IM (1963). Papillary lymphoid hyperplasia at the ileocaecal valve as a cause of acute intussusception in infancy. Arch Dis Child.

[11] Kokkonen J, Karttunen TJ (2002). Lymphonodular hyperplasia on the mucosa of the lower gastrointestinal tract in children: an indication of enhanced immune response?. J Pediatr Gastroenterol Nutr.

[12] Shteyer E, Koplewitz BZ, Gross E, Granot E (2003). Medical treatment of recurrent intussusception associated with intestinal lymphoid hyperplasia. Pediatrics.

[13] Jantchou P, Bonnin V, Aubert D (2008). Oral corticosteroids are efficient in recurrent intussusception associated with intestinal lymphoid hyperplasia. Arch Pediatr.

[14] Lopez R, Beasley SW, Blakelock R, Maoate K, McBride CA, Slade C (2012). The role of steroids in children with multiple recurrences of intussusception. J Paediatr Child Health.

[15] Ravitch MM, McCune RM (1950). Intussusception in infancy and children. J Pediatr.

[16] Koh CC, Sheu JC, Wang NL, Lee HC, Chang PY, Yeh ML (2006). Recurrent ileocolic intussusception after different surgical procedures in children. Pediatr Surg Int.

[17] Apelt N, Featherstone N, Giuliani S (2013). Laparoscopic treatment of intussusception in children: a systematic review. J Pediatr Surg.

[18] Zhao J, Sun J, Li D, Xu WJ (2022). Laparoscopic versus open reduction of idiopathic intussusception in children: an updated institutional experience. BMC Pediatr.

[19] Li SM, Wu XY, Luo CF, Yu LJ (2022). Laparoscopic approach for managing intussusception in children: analysis of 65 cases. World J Clin Cases.

[20] del-Pozo G, Albillos JC, Tejedor D, Calero R, Rasero M, de-la-Calle U, et al. Intussusception in children: current concepts in diagnosis and enema reduction. Radiographics. 1999;19(2):299–319. 10.1148/radiographics.19.2.g99mr14299 10194781

